# Invasive Bighead and Silver Carps Form Different Sized Shoals that Readily Intermix

**DOI:** 10.1371/journal.pone.0157174

**Published:** 2016-06-08

**Authors:** Ratna Ghosal, Peter X. Xiong, Peter W. Sorensen

**Affiliations:** Department of Fisheries, Wildlife and Conservation Biology, University of Minnesota, St. Paul, MN 55108, United States of America; Bournemouth University, UNITED KINGDOM

## Abstract

Two species of congeneric filter-feeding microphagous carps from Asia, the bighead and the silver carp, were recently introduced to North America and have become highly invasive. These species of carp have similar food habits but the silver carp has the unique habit of jumping when disturbed. Both species have complex but poorly understood social behaviors and while both are thought to aggregate (form groups) and shoal (form tight social groups), this possibility has not yet been examined in these species. The present study examined the grouping tendencies of these species in the laboratory and the effects of fish density and species identity on it. Using nearest neighbor distance (NND) as a metric, we showed that both juvenile bighead and juvenile silver carp grouped (aggregate) strongly (P<0.05) but to different extents, and that fish density had no effect (P>0.05) on this behavior. Within aggregations, bighead carp tended to form a single large shoal while silver carp formed shoals of 2–3 individuals. Further, when tested as mixed-species groups, bighead and silver carp readily shoaled with each other but not with the common carp, which is from Eurasia and a member of another feeding guild. Due to their similar feeding strategies, we speculate that the bighead and silver carp tend to aggregate and shoal to facilitate both their foraging efforts and to avoid predation, while the differences in the size of the shoals they form may seemingly reflect their different anti-predation strategies. These complex shoaling behaviors likely influence Asian carp distribution in rivers, and thus how they might be sampled and managed.

## Introduction

The bigheaded carps, which include the silver carp, *Hypophthalmichthys molitrix*, and the bighead carp, *H*. *nobilis*, evolved in large rivers in Asia. Both of these species (which are often considered a type of “Asian carp”) are microphagous and consume a wide and overlapping range of very small phyto- and zooplankton as well as detritus [1, 2, 3]. While bighead carp grow quickly to a very large size (80kg), silver carp only grow to 20kg but have a unique and striking tendency to jump up to 3 m when startled [4]. Jumping presumably reflects an anti-predation strategy in the silver carp [5]. Both species of Asian carp are important to global aquaculture and were introduced to North America in the 1970’s. However, both soon escaped and have since become highly abundant and invasive in the Mississippi River where they can comprise up to 75% of the fish biomass [4] and freely hybridize [6]. Anecdotal reports suggest that both juvenile and adult bigheaded carp tend to group, or aggregate, strongly [7,8] (Chapman DC, USGS, Columbia, Missouri; Phelps Q, University of Southern Missouri, Missouri, personal communication); however, this behavior has not, to the best of our knowledge, been systematically studied. An understanding of aggregation and shoaling behaviors would provide insight into their feeding ecology and predators as well as their patterns of distribution in open waters. Indeed, if these species shoal strongly this could explain why both adult and juvenile silver and bighead carp have proven very difficult to locate and sample using traditional trap-nets in stretches of rivers where they are still rare [9] because clustered items are typically more difficult to locate in lower densities [10].

Fish, including carp, can exhibit several types of grouping behaviors [11–13]. The simplest type of grouping behavior is typically termed an “aggregation” and it is often defined as a tendency to form a “non-random collection of individuals” [14]. Fish may aggregate for various reasons, which can be either social (i.e. for mutual benefit), or nonsocial (i.e. coincidental non-random distribution related to a patchy distribution of resources or habitat) [12, 15–17]. Because many species of fish are food-limited and compete for food, the tendency to aggregate can be affected by fish size and density as well as food availability [15, 16, 18].

Shoaling is a type of aggregation, which is characterized by consistently small nearest neighbor distances (NND) between fish. Fish may form shoals for several reasons that presumably benefit individual fitness [19, 20] and relate to the increased overall sensory awareness of coordinated groups which can enhance individuals’ abilities to locate food, avoid predators, find mates, and/or orientate [21–25]. Notably, many closely related species of fish also form mixed-species shoals. This phenomenon appears to be especially common amongst related species that have shared food preferences but is counter-balanced when food is very limiting [26–28]. For example, Ward et al [27] demonstrated that chub *Leuciscus cephalus* shoal equally with conspecifics and closely related heterospecifics (*Phoxinus phoxinus*) at low densities but when densities of the former increased (along with competition for food), the chub only shoaled with conspecifics [27]. Exceptionally tight shoals that entail synchronized swimming behavior are known as “schools” [11] which may reflect enhanced locomotor efficiencies in flowing water [25, 29] and/or increased anti-predator benefits due to organized collective motion and vigilance [30]. While aggregation and shoaling behavior has been systematically described in many fish, it has only been described in a few filter-feeding species that have omnivorous food habits such as the bigheaded carp, and these are all marine fish, which are found in clear waters [24, 25]. We hypothesize that generalist filter-feeders such as the bigheaded carp that live in turbid waters will come together in aggregations and form shoals to help them find patches of food and to avoid predation in large rivers.

In this study we tested the tendencies of silver and bighead carps to aggregate and shoal in laboratory tanks. We used juveniles in this initial proof-of-concept study because adult carp are extremely large and very difficult to maintain and study in the laboratory. We asked three questions: 1) Do juvenile silver and bighead carp aggregate and what effects does density have on this behavior? 2) If silver and bighead carp aggregate, do they also form shoals (tight social groups) and do they shoal in the same manners? 3) If these species shoal, do they also shoal with each other or with other carp such as the common carp? We used the common carp because this species is also omnivorous, but feeds on bottom food (i.e. it is in a different feeding guild) [31, 32] and does well in both the laboratory and Mississippi River (i.e. results could be transferred to the field).

## Materials and Methods

### Experimental animals

Juvenile silver and bighead carp were raised from wild stocks at the Columbia Environmental Research Center (U.S. Geological Survey, Columbia, MO, USA) and shipped to the University of Minnesota by air. Juvenile common carp were raised in ponds from wild stock (Osage Catfisheries Inc., MO, USA) and also shipped. Fish ranged in size from 5 to 10 cm (total length, TL) and had undeveloped gonads. In Minnesota, juvenile silver, bighead and common carp were held in separate 230-l flow-through circular tanks supplied with 21°C well water and maintained on a 16h:8h (L:D) photoperiod. Silver and bighead carp were fed an algae-based diet comprised of a 2:1 ratio of spirulina to chlorella as well as some dried flake food (Bulk foods, OH, USA) while common carp were fed pellets (Skretting, UT, USA). Feeding occurred in late evening and was to satiation. Experimental procedures were in compliance with the Guidelines of the University of Minnesota Animal Care and Use Committee (IACUC) and all necessary federal and state permits for shipping and holding prohibited species were obtained. All the fish were euthanized with MS-222 (Western Chemicals, Ferndale, WA, USA) after completion of the experiment.

### General methods

For testing, carp were transferred from their stock tanks and placed into 1.5 m circular observation tanks filled with 21°C well water to a depth of 20cm in the evening (between 16–18h) and before feeding. Fish were tested either as single-species groups of 4, 8, 12 and 20 fish to test the effects of density, or as mixed-species groups of 8 comprised of 4 silver carp along with 4 bighead carp or 4 common carp. Common carp were also tested as a single-species group of 8 fish or as part of the mixed-species experiment. Eight trials were conducted for single-species groups and 6 for the mixed-species groups. After testing, fish were returned to their home (stock) tanks and were given a rest period of at least 72 h, after which they were selected at random for reuse. Observation tanks were covered with dark plastic and had extremely dim (15W) red bulbs (4 bulbs for each tank) supplemented with overhead infrared lights that were run on a 16h:8h L:D photoperiod to match the stock tanks. These conditions simulated those of turbid rivers. For testing, fish were first acclimated overnight without being fed in observation tanks and the following day at 0900h (after lights were on), the water was shut off and 1 h later, fish behavior was recorded using an overhead low-light camera, which recorded to a DVD for 15-min.

Video recordings were later analyzed by playing back the images and manually measuring distances between fishes to the nearest cm using a ruler on a computer screen to determine nearest neighbor distances (NND). Measurements were based on that fish seen to be located closest to the 12 o’clock position of the computer screen (19” Dell Ultrasharp, model #1905-FP, TX, USA) and then moving to the nearest fish to its right following standard protocols [33]. In other words, we measured distances between fishes along a single chain by measuring the distance between each fish and then the fish on its right [33]. These distances were measured every 15-sec for each 15-min trial. All NND measurements were tested for normality (Shapiro-Wilk test) before further analysis was performed (see below) using the R statistics package (version 3.0.3).

### Statistical analysis of aggregation behavior

To test for aggregation in single-species groups, NND was used to calculate aggregation indices (R) for each group size following Clark and Evans [14]. Briefly, for this index, a value, R is calculated in which R = R_A_/R_E_, and R_A_ is the mean of the distances to nearest neighbors observed during the experiment (mean NND) while R_E_ is the mean distance to nearest neighbor expected in an infinitely large random distribution of a particular density. R_E_ is expressed as 1/2√ρ where ρ is the number of individuals per unit of area. A R value of 1 reflects no aggregation (i.e. a random distribution) while 0 reflects a single aggregation in which all fish occupy a single locus [14]. We calculated the R-value for every 15-sec interval for each 15-min trial and then expressed them as a mean ± S.D. These R-values were then analyzed in two steps. First, for each group size, the average R-value was compared to 1.0 using a one-sample t-test to determine if their distribution was random (or not). Second, we compared R-values of bighead to those of silver carps across different group sizes using two additional analyses. We used a multiple regression model to test for the overall effects of density (a continuous variable) and species on aggregation behavior. Additionally, we used a two-way ANOVA with Tukey’s post hoc tests to directly test for differences between species at each group size after adjusting for multiple comparisons. In addition, we also calculated aggregation indices (R) for common carp (group size 8) in the same manner.

To determine if fish formed mixed-species aggregations, R-values were calculated and analyzed in the same manner as for single-species using data from the group size of 8 fish. Briefly, R-values were averaged over the trials and the average R value was then compared to 1 using a one-sample t-test to determine if the distribution was random or not. Next, we conducted one-way ANOVA analysis on R-values of single- (bighead, silver and common carp) and mixed- species (bighead and silver, bighead and common, and silver and common carp) groups of 8 fish. A one-way ANOVA analysis was carried out to test the effects of species identity on aggregation behavior.

### Statistical analysis of shoaling behavior

Analysis of shoaling behavior was performed for group sizes of 8 fish using both the single- and mixed-species groups. We focused on this group size because it was the largest size we could evaluate and allowed us to include data from mixed-species groups. This is an extremely time-consuming analysis so we tested just one group size. To analyze shoaling behavior, we re-evaluated the NND measurements for each trial using the 3-body length shoaling criteria suggested by Pitcher and Parrish [34]. We assigned a value of 1 to each inter-fish measurement if NND was ≤3 body lengths and 0 if NND was >3 body lengths. Next, we calculated the number of 0’s (i.e. the number of breaks in an aggregation) and the average number of fish in each shoal at each 15-sec interval for each 15-min trial. Later, we calculated the number of breaks in each aggregation (ignoring single isolated fish) to describe the number of shoals within that aggregation. Lastly, we calculated the total number of single isolated fish at each 15-sec interval for each trial to provide another measure of shoal structure. The number of shoals, fish per shoal and the number of single isolated fish were then averaged across trials to express as a mean ± S.D. We compared the number of shoals, the number of fish per shoal and the number of single isolated fish using separate one-way ANOVA with Tukey’s post hoc tests across all the groups including bighead, silver, common, bighead and silver, bighead and common, and silver and common carp. Species identity was taken into consideration for trials that included common carp as common carp could be identified by their wider body contour. Evaluation allowed us to calculate the number of mixed-species shoals (shoals in which common carp occurred together with either silver or bighead carp) for every 15-sec in an entire 15-min trial. The number of mixed-species shoals were then averaged across all the trials and expressed as mean percentage ± S.D.

## Results

### Aggregation behavior

**Fig 1:**
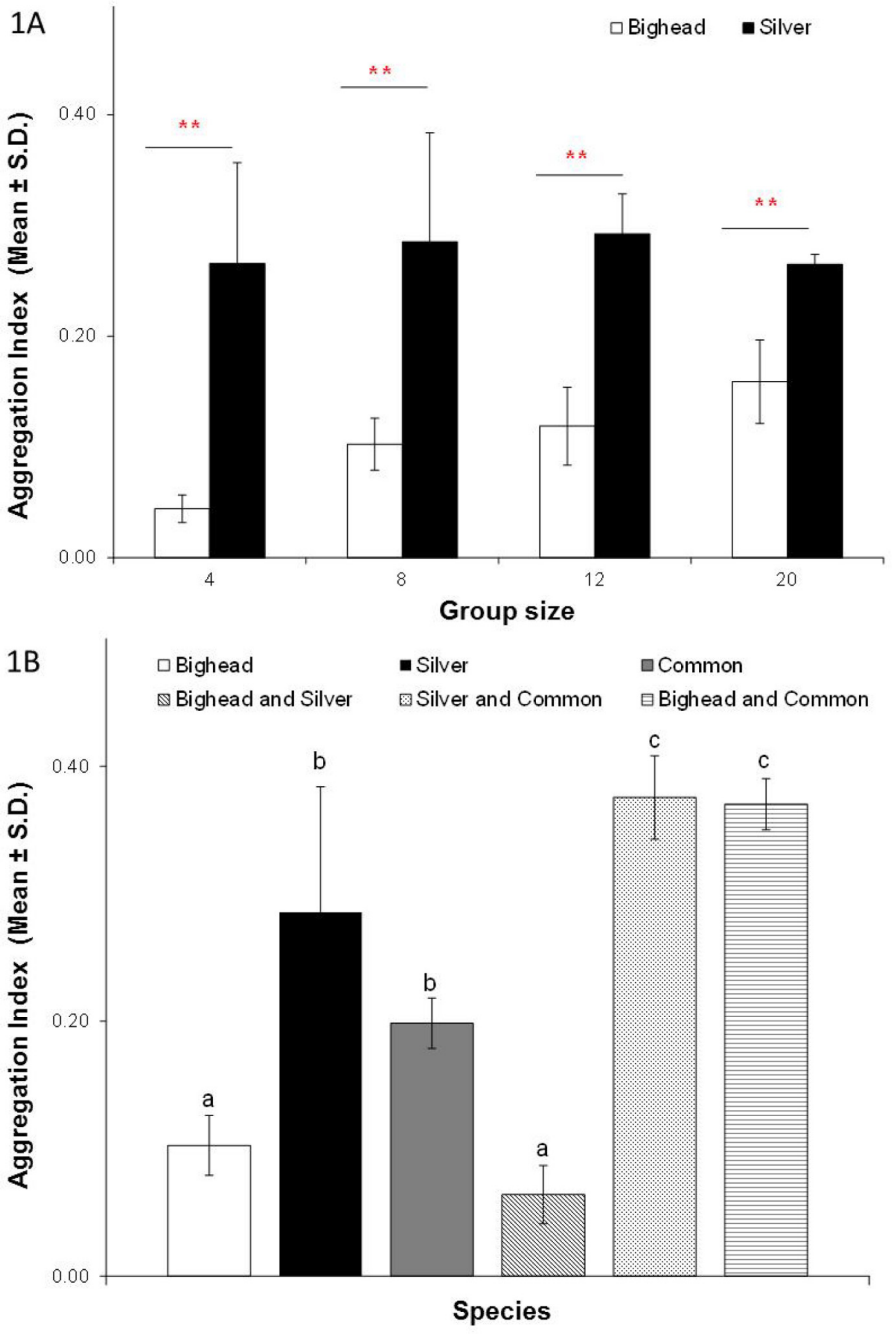
**A. Aggregation indices (Mean ± S.D.) of single-species groups—bighead (n = 8 trials) and silver (n = 8 trials) carp.** ** indicates P<0.05, two-way ANOVA with Tukey’s post hoc tests. **B. Aggregation indices (Mean ± SD) of single-species and mixed-species groups—bighead (n = 8), silver (n = 8), common (n = 6), bighead and silver (n = 6), silver and common (n = 6), bighead and common (n = 6)**. Different letters describe significant differences (P<0.05, one-way ANOVA with Tukey’s post hoc tests) between groups. Values for single-species groups of bighead and silver were replotted from Fig 1A.

Both bighead and silver carp aggregated when tested as single-species groups and at all densities (i.e. their R-values were different from 1, one sample t-test, P<0.05; t scores for bighead carp: t [df = 7] = 271.5 for the group size of 4; t [df = 7] = 254.5 for the group size of 8; t [df = 7] = 62.2 for the group size of 12; t [df = 7] = 59.3 for the group size of 20; t scores for silver carp: t [df = 7] = 22.9 for the group size of 4; t [df = 7] = 20.0 for the group size of 8; t [df = 7] = 50.2 for the group size of 12; t [df = 7] = 209.3 for the group size of 20). All NND measurements were normally distributed (Shapiro Wilk test, P>0.05) and a two-way ANOVA of R-values of bigheaded carps indicated a significant effect of species (F_1,56_ = 99.39, P<0.05), no effect of density (F_3,56_ = 2.79, P>0.05), and no interaction (F_3,56_ = 1.66, P>0.05) (Fig 1A). Multiple regression analysis (R^2^ = 0.6, P<0.05) described similar results as the ANOVA with a significant effect of species (t = 5.2, P<0.05), no effect of density (t = 2.4, P>0.05) and no interaction (t = -1.5, P>0.05) (Table 1). For each of the group sizes, R-values were different between silver and bighead carp (P<0.05, two-way ANOVA with Tukey’s post-hoc tests) (Fig 1A). Bighead carp had lower R-values (range: 0.04–0.1) than silver carp (range: 0.27–0.29) (P<0.05, Tukey’s post hoc tests), indicating they formed tighter aggregations (Fig 1A). Common carp also aggregated, with their R-values being different from 1 (one sample t-test, P<0.05, t [df = 7] = 113.1) (Fig 1B).

**Table 1:** Summary of a multiple regression model showing effects of density and species on aggregation index (R).

lm(formula = R ~ Density *Species)^+^				
	Estimate	Std. Error	t value	Pr(>|t|)
(Intercept)	0.042439	0.026680	1.591	0.11752
Density	0.004117	0.002136	2.499	0.1048
Species	0.216476	0.041021	5.277	2.38e-06 ***
Density:Species	-0.005097	0.003298	-1.546	0.12802

^+^Details of model: Residual standard error: 0.07149 on 61 degrees of freedom; Multiple R-squared: 0.6003, Adjusted R-squared: 0.5781; F-statistic: 27.03 on 3 and 61 DF, p-value: 8.235e-11

***P<0.05

All mixed-species groups also aggregated (i.e. their R values were different from 1, one sample t-test, P<0.05; t [df = 7] = 132.9 for bighead and silver carp; t [df = 7] = 58.4 for silver and common carp; t [df = 7] = 89.0 for bighead and common carp). One-way ANOVA showed significant effects of species identity on R-values (F_5,36_ = 7.4, P<0.05) (Fig 1B). Tukey’s post-hoc tests showed that the R-values were lowest (range: 0.04–0.06) in the single-species aggregation of bighead carp as well as the mixed-species aggregation that included both bighead and silver carp, indicating tighter groupings for these species combinations.

### Shoaling behavior

**Fig 2:**
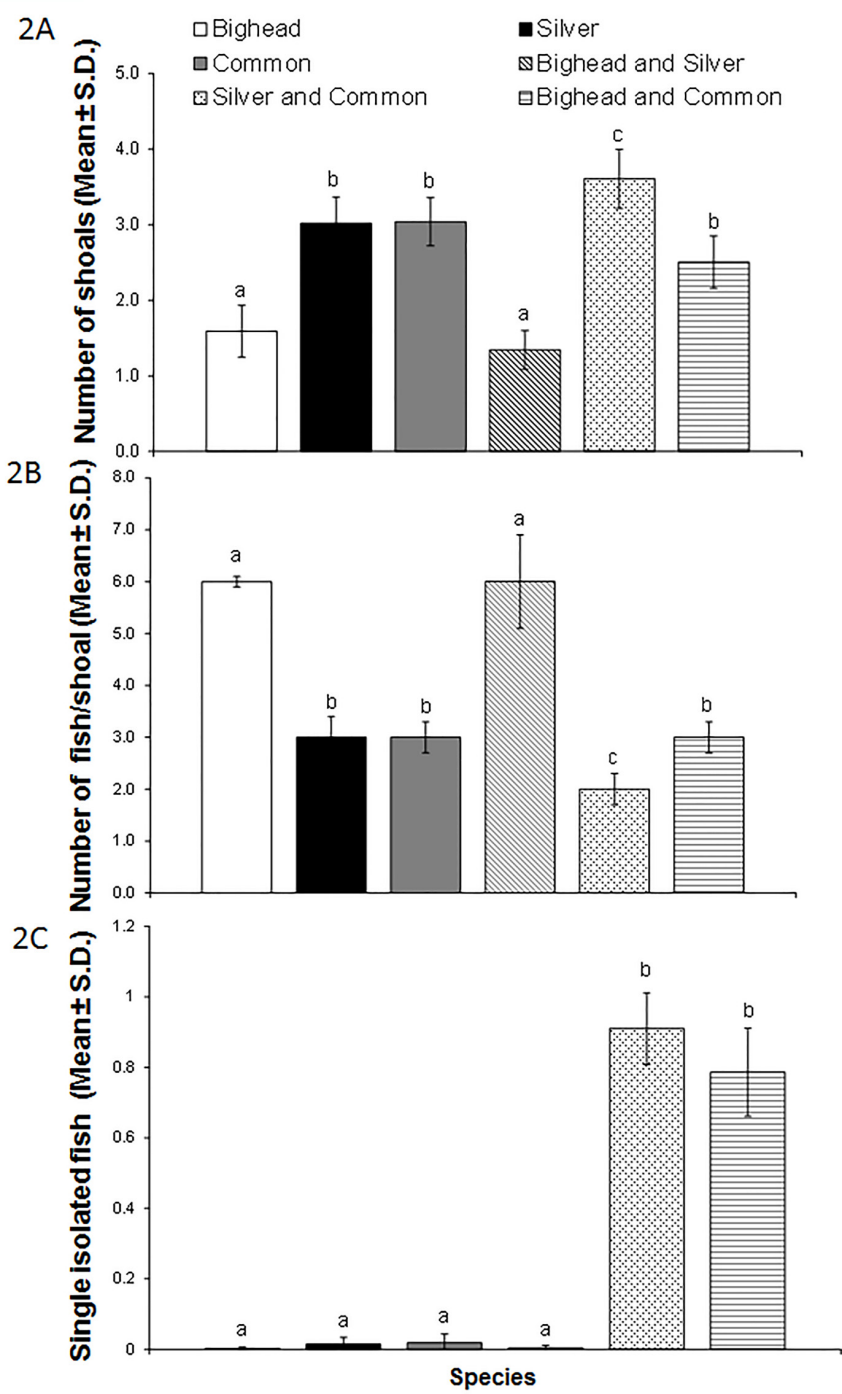
**A. Number of shoals (Mean ± SD); B. Number of fish/shoal (Mean ± SD); C. Single isolated fish (Mean ± SD) in single- and mixed-species groups—bighead (n = 8), silver (n = 8), common (n = 8), bighead and silver (n = 6), silver and common (n = 6), bighead and common (n = 6).** Different letters indicate significant differences (P<0.05, one-way ANOVA with Tukey’s post hoc tests). The decimal values in the mean number of fish/shoal were rounded to nearest whole number.

All three species of carp formed shoals but the number and the size of these shoals differed by species (P<0.05). One-way ANOVA analysis to test for the effects of species on the number of shoals across the 6 groups (bighead, silver, common, bighead and silver, bighead and common, silver and common carp) showed significant effects (F_5,36_ = 34.45, P<0.05) (Fig 2A). A Tukey’s post-hoc test showed that single-species aggregations of bighead carp formed fewer (an average of 1.5 shoals) but larger shoals (an average of 6 fish, see below) than did silver carp, which formed several smaller shoals containing approximately 3 fish (P<0.05). Mixed-species groups of bighead and silver carp behaved in an identical fashion to bighead carp alone (P>0.05), and often formed a single mixed-species shoal (Fig 2A). This was not the case for mixed-species groups of common carp with either bighead or silver carp. Mixed-species aggregations of bighead and common carp formed an average of just over 2 shoals and those of silver and common carp formed an average of over 3 shoals (P<0.05, Tukey’s post-hoc test) (Fig 2A).

One-way ANOVA analysis to test for the effects of species identity on shoal size across the 6 groups of bighead, silver, common, bighead and silver, bighead and common, and silver and common carp, also showed significant effects that paralleled the results of the shoal number analysis (F_5,36_ = 6.559, P<0.01) (Fig 2B). Tukey’s post-hoc tests showed that single-species aggregations of bighead and mixed-species aggregations of bighead and silver carp formed the largest shoals, with an average of 6 fish per shoal (Fig 2B). Single-species aggregations of silver carp, common carp and mixed-species aggregations of bighead and common carp formed an average shoal size of 3 fish (Fig 2B). Mixed-species aggregations of silver and common carp formed the smallest size shoals (P<0.05, Tukey’s post hoc test), with an average of 2 fish (Fig 2B). The mean percentages of mixed-species shoals (shoals in which common carp occurred with either silver or bighead carp) were 22 ± 1.3 for mixed-species groups of bighead and common carp (i.e. 78% of the shoals were just single-species) and 18 ± 2.1% for mixed-species groups of silver and common carp (i.e. 82% of the shoals were single-species). Finally, one-way ANOVA analysis to test for the effects of species identity on the number of single isolated fish in groups showed significant effects (F_5,36_ = 135.5, P<0.01) (Fig 2C) with the number of single isolated fish being highest in mixed-species groups containing either silver and common carp or bighead and common carp.

## Discussion

This study found that juvenile bighead and silver carp aggregated strongly and formed shoals, but in very different ways and that density had no influence on aggregation behaviors. Juvenile bighead carp formed tighter aggregations and larger shoals, while the silver carp formed smaller shoals of only 2–3 fish. Interestingly, when these species were placed together, they shoaled together forming single, large shoals that were not different than those formed by just bighead carp. However, both bighead and silver carp did not shoal consistently with the common carp and single, isolated fish were often present in mixed-species aggregations. The aggregation behavior and shoaling tendencies of bighead and silver carp are presumably adaptive, imparting advantages to both food search and avoiding predation [15, 20, 21]. It is possible that silver carp form smaller shoals than bighead carp because individual silver carp can also avoid predation by jumping. Instead of being randomly distributed, bigheaded carps occur in shoals or clusters that may make them hard to locate in large bodies of water, particularly in the stretches of river where they occur in low numbers [10]; however, this attribute could perhaps also be exploited to control and manage them better.

The most important finding of this study is likely that juvenile bighead, silver and common carps, like many juvenile fish, aggregate and shoal strongly [15]. Although fish density did not appear to influence aggregation, this possibility needs to be evaluated using larger group sizes. Bigheaded carps may aggregate and shoal to both efficiently locate their microscopic, planktonic food and to avoid predators [15, 20, 21]. Food search and consumption is presumably very energetically demanding in both silver and bighead carp, which use a specialized type of filter-feeding that involves buccal pumping [3]. We speculate that juvenile bighead and silver carp find it easier to locate patches of planktonic and microscopic particles in turbid river water by forming shoals and aggregations, and assessing each other’s feeding responses [35]. Juvenile common carp may derive similar benefits from aggregating and shoaling as they search for a wide range of food items in bottom sediments, which can also be patchy.

Somewhat surprisingly, this study also found that two congeneric bigheaded carps, which have very similar microphagous feeding habits, shoaled in very different manners. While groups of 8 juvenile silver carp tended to form several small shoals of 3 fish, groups of 8 juvenile bighead carp tended to form single large shoals of 6–7 fish. Even larger shoals of bigheaded carps have been anecdotally described in large laboratory tanks containing hundreds of fish (Dr. Jan Hoover, U.S. Army Corps of Engineers, Vicksburg, MS). We speculate that these differences in shoaling behaviors may be related to the fact that only silver carp jump and that this attribute may reflect a unique anti-predation strategy. Thus, both species aggregate and shoal to find food efficiently with non-jumping bighead carp forming very large shoals as these groupings can also shield individuals from predation [34, 36]. Alternatively, silver carp may form smaller groups because individual silver carp can jump to avoid predators [37]. However, this strategy appears to change in mixed species groups as described below. It would be very interesting to test this hypothesis in presence of food resources and predators.

Another novel and important finding of this study was our observation that bighead and silver carp aggregate and shoal together in tight mixed-species groups. So tight were these mixed-species groups that they did not differ from those of bighead alone (but did differ from those of silver carp alone). Strikingly, neither bighead nor silver carp shoaled consistently with the common carp and isolated individuals were consistently observed in these aggregations. Notably, the common carp has very different feeding habits (benthic vs. surface feeding) and is in a different feeding guild than the bigheaded carps [8, 31, 32]. Since our observations were performed in near darkness, these specific-specific tendencies were likely mediated by species-specific pheromones, which have already been shown to be used by other carp species like the common carp and the goldfish, *Carassius auratus* [38]. Notably, several closely related species with overlapping feeding habits such as the bigheaded carps [39] are also known to participate in mixed-species shoals [26–28, 40]. Our results suggest that juvenile silver carp may have developed a tendency to aggregate and shoal with bighead carp to gain access to food and to avoid predation by living amongst larger groups of bighead carp. Future work on the shoaling tendencies of the bigheaded carps in the wild is now required. It would also be interesting to test whether the silver and /or bigheaded carp aggregate with other naturally sympatric, or non-sympatric filter feeders like the gizzard shad (*Dorosoma cepedianum*), which have similar feeding habits.

Our results have important implications for fisheries management. In particular, our findings suggest that monitoring schemes for these species should consider the possibility that juvenile bigheaded carp is unlikely to be randomly distributed and that mixed species shoals may also be common. Fisheries managers presently use trap-nets at fixed locations to capture these fish [9], which they are unlikely to intercept and catch if these fish are not randomly distributed and are only present as a few shoals. This might be especially true in parts of the river where densities are very low [9]. Conversely, techniques that exploit the shoaling behaviors of these fish, such as the Judas fish technique [41, 42], in which a few individuals are marked and tracked as they locate others, now warrant serious consideration for sampling and removal. This would be especially valuable for adult carp. Anecdotal observations indicate that adult bighead carp also shoal [9] but this possibility now clearly warrants further study. The possibility that adult bighead and silver carp shoal together could also help explain the extensive hybridization between the two in the Mississippi River [6]. However, while numerous species of fish have already been shown to exhibit similar types of grouping and shoaling behaviors in the field as in the laboratory [22], explicit tests of the shoaling tendencies of both juvenile and adult wild bigheaded carp should now be performed.

In conclusion, our proof-of-concept laboratory study clearly demonstrates that juvenile bighead and silver carp aggregate and shoal in the laboratory, but in very different ways that seemingly reflect shared food habits [26–28] but different anti-predation strategies [37]. Mixed-species shoals of both juvenile and adult bigheaded carps may be common in the natural environment and could be exploited. Future studies should now address the effects of habitat, food availability, the presence of predators, and maturity on shoaling in the carps. We hope our study might stimulate this important work.
